# The Molecular Role of IL-35 in Non-Small Cell Lung Cancer

**DOI:** 10.3389/fonc.2022.874823

**Published:** 2022-05-26

**Authors:** Yuqiu Hao, Hongna Dong, Wei Li, Xuejiao Lv, Bingqing Shi, Peng Gao

**Affiliations:** Department of Respiratory Medicine, Second Hospital of Jilin University, Changchun, China

**Keywords:** NSCLC, IL-35, Treg, PD-1/PD-L1, Th17

## Abstract

Non-small cell lung cancer (NSCLC) is the most common type of lung cancer and a common cause of cancer-related death. Better understanding of the molecular mechanisms, pathogenesis, and treatment of NSCLC can help improve patient outcomes. Significant progress has been made in the treatment of NSCLC, and immunotherapy can prolong patient survival. However, the overall cure and survival rates are low, especially in patients with advanced metastases. Interleukin-35 (IL-35), an immunosuppressive factor, is associated with the onset and prognosis of various cancers. Studies have shown that IL-35 expression is elevated in NSCLC, and it is closely related to the progression and prognosis of NSCLC. However, there are few studies on the mechanism of IL-35 in NSCLC. This study discusses the role of IL-35 and its downstream signaling pathways in the pathogenesis of NSCLC and provides new insights into its therapeutic potential.

## Introduction

Lung cancer (LC) is one of the deadliest cancers worldwide. In 2018, a global report suggested high incidence and mortality associated with LC, and important impact of LC on global health problems (1). It is classified into small-cell LC (SCLC, approximately 15% cases) and non-small-cell LC (NSCLC, approximately 85% cases) (2). Its etiology is multifactorial, and pathogenesis is incompletely understood. Available literature reveals that dysregulated inflammatory responses increase the risk of chronic diseases and cancers, including LC (3). Anti-inflammatory cytokines play an active role in reducing tumor growth, metastasis, apoptosis, and angiogenesis (4). Thus, studies evaluating LC pathogenesis and molecular mechanisms can provide a basis for identifying new biomarkers and developing targeted therapies.

LC and anticancer treatment result in airway obstruction and opportunistic infections, thereby increasing the morbidity and mortality (5, 6). Additionally, presence of febrile neutropenia in LC patients receiving myelosuppressive chemotherapy has the risk of bacterial infections. Thus, early diagnosis and treatment of these infections is crucial to improve the prognosis (7).

Interleukin 35 (IL-35) is a newly discovered member of the interleukin family and has been reported to have anti-inflammatory and immunoregulatory properties (8–10). Structurally, it is a heterodimer comprising of two subunits, EBI3 and IL-12p35 (11–14). Additionally, IL-35 receptor includes two subunits, IL-12Rβ2 and glycoprotein 130 (gp130). IL-35 mediates signaling through signal transducer and activator of transcription (STAT) 4, STAT1, and STAT4/STAT1 in the presence of subunits IL-12 Rβ2, gp130, and IL-12Rβ2/gp130, respectively (15).

IL-35 is overexpressed in prostate cancer (16), LC (17), gastric cancer (18), and hepatocellular carcinoma (19), and its overexpression is directly implicated in tumor progression and poor prognosis of LC (20), pancreatic cancer (21), hepatocellular carcinoma (22), breast cancer (23), renal cell carcinoma (24), laryngeal squamous cell carcinoma (25), osteosarcoma (26), and colorectal cancer (27). IL-35 levels are positively correlated with tumor stage (tumor size, metastasis to adjacent lymph nodes, and distant metastases) in pancreatic ductal adenocarcinoma (28), breast cancer (29, 30), acute myeloid leukemia (31), prostate cancer (32, 33), and colorectal cancer (27). Additionally, it promotes tumor growth as well as immune escape, and can be used as a prognostic indicator (10, 27, 28).

IL-35 limits anti-tumor immunity in the tumor microenvironment by regulating T cell responses (16, 34, 35). In breast cancer, it promotes tumor progression by inducing the conversion of conventional T cells to inducible Tregs (iTr35) (30). Additionally, it can recruit Treg cells in colorectal cancer (27), induce the accumulation of CD11b^+^ Gr1^+^ myeloid cells in the tumor microenvironment (36), stimulate angiogenesis, and reduce the infiltration of activated CD8^+^ T cells into the tumor (10).

## Study on the Mechanism of Action of IL-35 in Various Diseases

In the tumor microenvironment, the relationship between inflammation and the immune system is very complex. The role of IL-35 *in vivo* (**Table 1**) and *in vitro* cellular level (**Table 2**) with autoimmune diseases, allergic diseases, and tumors has been extensively studied. It is an anti-inflammatory factor that inhibits Th2-type cytokine production in allergic rhinitis and asthma and reduces eosinophilic airway inflammation (37, 50). In systemic lupus erythematosus (SLE) (42), inflammatory bowel disease (45), collagen-induced arthritis (CIA), psoriasis (48), autoimmune uveitis (39), and other autoimmune diseases, IL-35 is involved in the development of disease by regulating the expression of inflammatory factors and immune response. In sepsis, it exerts anti-apoptotic and inflammatory effects (46). In sarcoidosis, it is associated with the inflammation of loose nodular granulomas, as well as increased Breg and decreased Treg in peripheral blood (38). In acute kidney injury, it exerts anti-inflammatory effects by decreasing the secretion of pro-inflammatory factors (47). Contrarily, in acute respiratory distress syndrome (ARDS), IL-35 appears to be protective, and lung injury is a result of its neutralization (44).

**Table 1:** The role of IL-35 in disease mouse models.

Disease	Regent	Molecular target	Function	Reference
Allergic rhinitis	IL-35	↓IL-25, IL-33, TSLP, Eotaxin-1, Eotaxin-2, Eotaxin-3	Suppress Th2, ILC2, and eosinophilic inflammation	(37)
Sarcoidosis	IL-35mAb	↓Breg, ↑Treg	Promote loose granulomata	(38)
Autoimmune uveitis	i35-Exosomes	↑Treg	Anti-inflammatory	(39)
DNP	rIL-35	↓JNK, ↑JAK2, ↑STAT6	Promote microglial M2 polarization, anti-inflammatory, anti-apoptotic	(40, 41)
SLE	IL-35	↑JAK2, ↑STAT1, ↑STAT4, ↓STAT3, ↓MAPK	Anti- inflammatory	(42)
CIA	IL-35	↓iNOS, ↓ COX-2, ↓CCR7, ↑ CD206	Induce FLS apoptosis, promote M2 polarization, anti- inflammatory	(43)
ARDS	Anti-IL-35 EBI3/anti-IL-12 p35	↓CD4+/Treg ratio	Promote lung damage	(44)
Colitis	IL-35	↑IL-10, ↓IL-6, ↓IL-17, TNF-α, ↓macrophages, ↓T cell infiltration, ↑Treg	Anti-inflammatory	(45)
Sepsis	pIL-35	↑STAT1, ↑ STAT4, ↓ICAM-1, ↓ VCAM-1, ↓ IL-6, ↓ IL-8, ↑IL-10	Anti-inflammatory, antiapoptotic	(46)
Acute kidney injury	pIL-35	↓NF-kB, ↓TNF-α, ↓IL-1β, ↓IL-6	Anti-inflammatory	(47)
Psoriasis	pIL-35	↓pro-inflammatory factors, ↑IL-10, regulate M1/M2 macrophages, decrease the number of macrophages	Anti-inflammatory	(48)
Prostate cancer	rIL-35	↑Treg, ↓CD4+ and CD8+ T, promotes proliferation of MDSCs and promotes angiogenesis	Promote tumor progression	(16)
Heart transplant model	IL-35-MSCs	↓Th17, ↑CD4^+^ Foxp3^+^ T, ↓Th1/Th2	Regulate immune tolerance	(49)
Asthma	IL-35	↓Th2, ↓eosinophil counts, ↓formation of inflammatory DC	Anti-inflammatory	(50)

AKI, acute kidney injury; ARDS, acute respiratory distress syndrome; CIA, collagen-induced arthritis; DNP, diabetic neuropathic pain; FLS, fibroblast-like synoviocyte; ILC2, II innate lymphoid cells; MDSCs, myeloid-derived inhibitory cells; pIL-35, plasmid-IL-35; rIL-35, recombinant IL-35; rhIL-35, recombinant human IL-35; SLE, systemic lupus erythematosus; TSLP, thymic stromal lymphopoietin. ↑Increased; ↓Decreased.

**Table 2:** Functional study of IL-35 at cellular level *in vitro*.

Stimulus	Cell line	Regent	Molecular targets	Effect	Reference
Dermatophagoides pteronyssinus, Aspergillus fumigatus	HNECs	IL-35	↓ IL-25, IL-33, TSLP, eotaxin-1, eotaxin-2, eotaxin-3	Regulation of Th2, ILC2, and eosinophilic inflammation	(37)
HDM	PBMCs	rIL-35	↑ MEK, ↑ JNK, ↓ IL-17, ↓IL-23	Inhibit Th17 response	(51)
LPS,IFN-γ,IL-4	Hepatocellular carcinoma, THP-1	rhIL-35	↑ N-cadherin, ↑ E-cadherin, ↑STAT3	Promote EMT and MET	(52)
–	Mesangial cells	IL-35	↑ JAK2, ↑ STAT1, ↑ STAT4, ↓ STAT3, ↓ MAPK, ↓TNF-α, ↓ IL-6, ↓ IL-17A	Anti-inflammatory	(42)
TNF-α	PBMCs, FLS	IL-35	↓ iNOS, ↓ COX-2, ↓ CCR7, ↑ CD206	Induction FLS apoptosis, promote M2 polarization, anti- inflammatory	(43)
LPS	HUVECs	rhIL-35	↑ STAT1, ↑ STAT4, ↓ ICAM-1, ↓ VCAM-1, ↓ IL-6, ↓ IL-8, ↑ IL-10	Anti-inflammatory and antiapoptotic	(46)
TNF-α	Human bronchial epithelial cells	IL-35	↓ MUC5AC, ↓ ICAM-1, ↓ IL-6, ↓IL-8,↓ MCP-1, ↓ p38MAPK	Anti-inflammatory, inhibit pyroptosis and cell damage	(53)
–	Pancreas cancer cell	IL-35	Promote proliferation and inhibit apoptosis	Promote tumor development	(21)

COX-2, Cyclooxygenase-2; EMT, Epithelial-mesenchymal transition; FLS, Fibroblast-like synoviocyte; HNECs, human nasal epithelial cells; HUVECs, human umbilical vein endothelial cells;ICAM-1, Intercellular adhesion molecule-1; IFN-γ, Interferon-gamma; iNOS, Inducible nitric oxide synthase; JNK, Jun N-terminal kinase; LPS, Lipopolysaccharide; MAPK, Mitogen-activated protein kinases; MET, Mesenchymal-epithelial transition factor; PBMCs, Peripheral blood mononuclear cells; pIL-35, Plasmid-IL-35; rIL-35, Recombinant IL-35; rhIL-35, Recombinant human IL-35; TSLP, Thymic stromal lymphopoietin; TNF-α, Tumor Necrosis Factor alpha; VCAM-1, Vascular cellular adhesion molecule-1. ↑Increased; ↓Decreased.

In liver cancer, IL-35 induces epithelial-mesenchymal transition (EMT) and mesenchymal-epithelial transition factor (MET) in macrophages with different polarization states, and promotes tumor progression (52). In pancreatic cancer, it promotes cell proliferation and inhibits apoptosis (21). In prostate cancer, it increases Treg expression, promotes proliferation of myeloid-derived inhibitory cells (MDSCs), angiogenesis, and tumor progression and inhibits CD4^+^ and CD8^+^ T lymphocyte levels (16).

## IL-35 and NSCLC

IL-35 expression is increased in the serum and tumor tissue of NSCLC patients (17) and in bronchoalveolar lavage fluid (BALF) and serum of NSCLC patients undergoing immunotherapy (54), demonstrating that this cytokine can serve as a therapeutic target for NSCLC. The study by Zhang et al. showed that plasma IL-35 levels in NSCLC patients were significantly higher than those in healthy controls (55). Additionally, the overexpression of IL-35 was significantly correlated with prognostic factors such as T stage, lymph node metastasis, micro-vessel density, and tumor differentiation, and total survival time increased in patients with low expression of IL-35 (55). A recent study by Li et al. showed that plasma IL-35 in the stage IV NSCLC patients was higher than that of the healthy group, and its expression levels were higher in the cachexia group than that of the non-cachexia group. Another study demonstrated that IL-35 was significantly associated with skeletal muscle atrophy (56). This was further confirmed in a mouse model that elevated IL-35 levels can induce skeletal muscle atrophy and cachexia (56). Overall, IL-35 is a key regulator of the development and prognosis of NSCLC. Additionally, a study involving surgically managed NSCLC patients demonstrated that compared with healthy controls, serum IL-35 levels were increased in patients with lung adenocarcinoma (ADC) and decreased in patients with lung squamous cell carcinoma (SCC). ADC patients had increased IL-35-expressing cells in tumor areas compared to corresponding tumor-free control areas. In SCC patients, there was also a trend of increased IL-35 in the tumor region, but this did not reach statistically significant level (17). The CD4 mRNA in the tumor region of ADC and SCC patients is reported to be lower than that of the control group and peritumoral region, respectively (17). It is suggested that IL-35 exerts an immunosuppressive effect by inhibiting CD4^+^ T cell-mediated immune responses, and ADC is more immunosuppressive than SCC (17). Furthermore, toll-like receptor 4 promotes the expression of histone lysine demethylase 3A (KDM3A) in lung ADC cell line, KDM3A interacts with forkhead box protein 3 (Foxp3) and promotes the secretion of immunosuppressive factors such as IL-35, which promotes immune escape of lung ADC (57). While IL-35 may not affect the survival and death of lung cancer cells (17), it may be involved in the pathological process of the NSCLC by regulating the microenvironment and immune response. Contrarily, an *in vitro* study demonstrated that IL-35 overexpression inhibits cancer growth by promoting apoptosis and inducing cell cycle arrest (58). The discrepancy in the results between tumor types may be because IL-35 expression depends on tumor type, stage, and microenvironment. Further studies are required to elucidate the underlying mechanism of IL-35 in NSCLC. This study discusses the molecular role of IL-35 in NSCLC.

## IL-35 and Tregs in NSCLC

CD4^+^ T cells participate in anti-tumor immunity and prevent immune escape by regulating the immune response. According to the pattern of cytokine secretion, these cells are classified into Tregs and T helper types 1 (Th1), 2 (Th2), and 17 (Th17) (59–63). IL-35 is secreted by Treg cells and inhibits T cell proliferation and function (8, 11, 35). Tregs promote tumor growth by suppressing host immune responses and promoting immune escape *via* the expression of transforming growth factor β, IL-10, and IL-35 (64–67). The increase in Tregs promotes tumor recurrence and reduces survival, thereby worsening prognosis (22, 68, 69). In NSCLC patients with checkpoint inhibitor pneumonitis (CIP), IL-35 expression increases the number of Treg and Th1 cells and the Th1/Th2 ratio (54). The number of Treg cells is increased in NSCLC (70, 71) and is closely linked with clinical stage and prognosis (72). IL-35 improves the ability of Tregs to induce immunosuppression and help prevent diseases (8, 73, 74). It participates in the pathogenesis of colorectal cancer by increasing Tregs and recruiting these cells to the tumor site (27). Currently, the complex mechanism of immune escape mediated by Treg cells is incompletely understood. Thus, further understanding of the role of IL-35 and Tregs in NSCLC may provide new insights into NSCLC treatment.

## IL-35, Th17, and Th17/Treg Imbalance in NSCLC

Some immunological studies in NSCLC have focused on Th1 and Th2 cells, and related factors (75–78). Th2 cytokines are increased, while Th1 cytokines are decreased in the peripheral blood of NSCLC patients compared with healthy controls (75), and the Th1/Th2 ratio is negatively correlated with LC prognosis (79).

Th17 regulates the expression and secretion of IL-17 and other cytokines and participates in tumor pathogenesis (80, 81). Additionally, both Th17 and IL-17 play a fundamental role in LC immunity (82–84), and have an anti-tumor or pro-tumor effect depending on the type of cancer (85, 86). The reasons for this paradox are unclear and require further investigation. IL-17 promotes the growth of NSCLC by inducing tumor cell proliferation (87), blood vessel formation (88, 89), lymphangiogenesis (90), and tumor invasiveness. Serum IL-17 levels are increased in NSCLC patients and are an independent prognostic factor (91). Th17 cells exert anti-tumor effects indirectly by recruiting CD8^+^ T cells and other immune cells (92).

Imbalances between Treg and Th17 cells occur in NSCLC. The number of these cells, and Foxp3 and RORγt expression are reported to be higher in the peripheral blood of NSCLC patients than in healthy controls. The number of Th17 cells is inversely correlated with the number of Tregs (93). The TregFoxp3^+^/Th17 ratio is valuable for diagnosing NSCLC and increases with increase in tumor stage (84).

IL-35 promotes tumor immune escape by increasing the number of IL-35producing iTr35 cells (30, 34, 73, 94). It also plays an immunosuppressive effect by promoting Treg cell proliferation and inhibiting Th17 cell differentiation (11, 95). These findings suggest that IL-35 is closely linked to Treg and Th17 cells in NSCLC; however, the mechanisms underlying this correlation remain unclear.

## IL-35 and PD-L1/PD-1 in NSCLC

**Figure 1:**
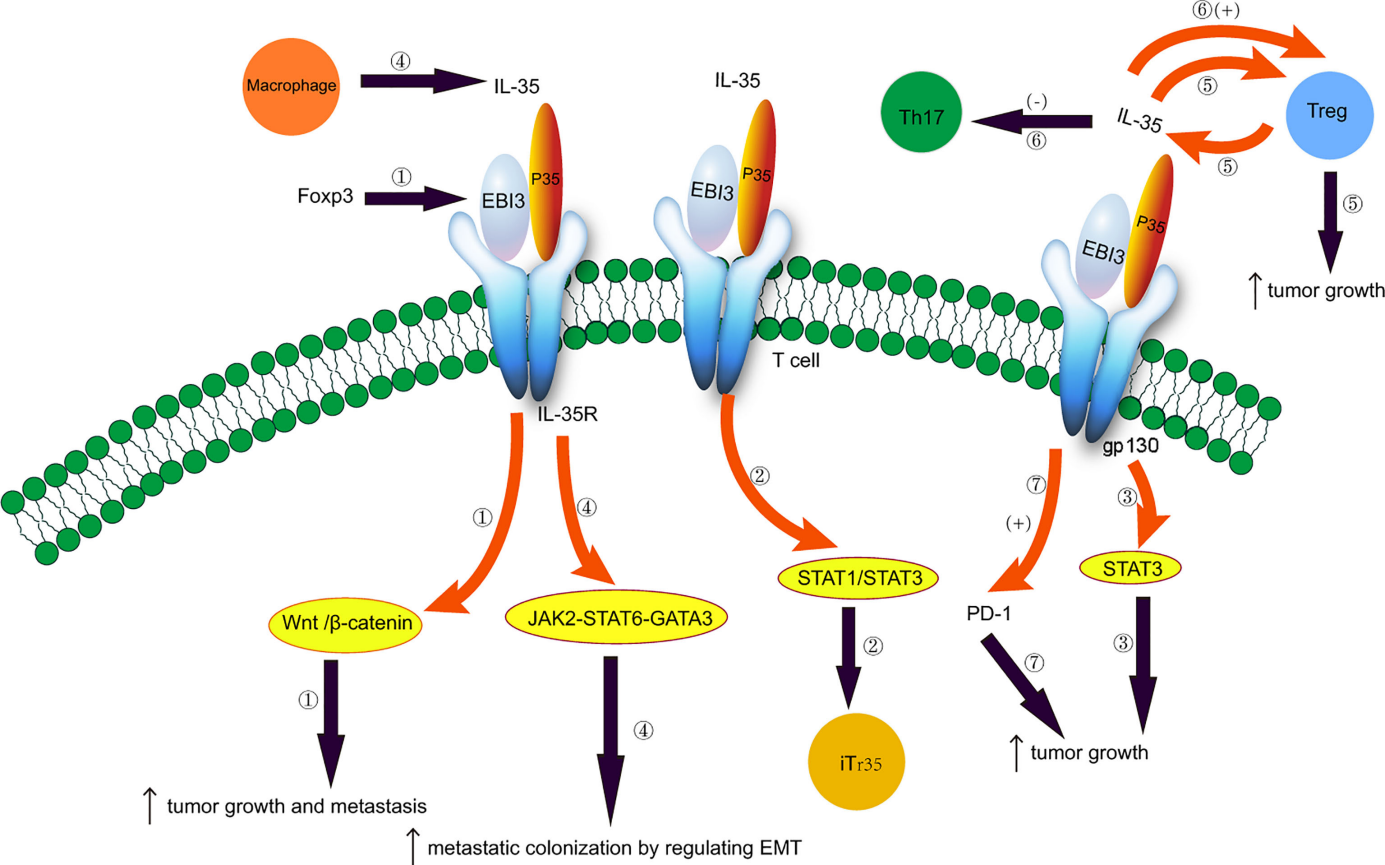
Potential role of IL-35 in non-small cell lung cancer. 1. Foxp3 targets the EBI3 subunit of IL-35 and induces tumor growth and metastasis by activating the Wnt/β-catenin signaling pathway. 2. IL-35 induces the conversion of conventional T cells to iTr35 through the STAT1/STAT3 pathway. 3. EBI3 promotes tumor growth through the gp130-STAT3 signaling. 4. Tumor-associated macrophages secrete IL-35 and promote metastatic colonization by regulating epithelial-mesenchymal transition through the JAK2-STAT6-GATA3 signaling pathway. 5. IL-35 is secreted by Treg cells, and IL-35 produced by cancer cells recruits Treg cells and induces tumor growth. 6. Imbalances between Treg and Th17 cells. IL-35 plays an immunosuppressive effect by promoting Treg proliferation and inhibiting Th17 cell differentiation. 7. IL-35 produced by Treg cells induces the expression of PD-1 and other inhibitory cytokines, impairing T cell function in the tumor environment and promoting tumor growth.

Therapeutic monoclonal antibodies targeting programmed cell death protein 1 (PD-1) or programmed cell death protein ligand 1 (PD-L1) can effectively treat NSCLC (96–98). PD-1 is expressed in B cells, T cells, and natural killer T cells (NK) (99). The role of PD-1/PD-L1 in CD8+ T cell failure has been elucidated (100–103). Anti-PD-1 therapy induces the expansion of specific subsets of exhausted CD8^+^ T cells that infiltrate the tumor (104), and inhibits CD8^+^ T cell-mediated tumor growth (105). The interaction between PD-1 and PD-L1 reduces the susceptibility of tumor cells to T cell cytotoxicity (106–108). The number of IL-35^+^ Foxp3^+^ T cells is positively associated with the number of thyroid transcription factor 1^+^ PD-L1^+^ cells in NSCLC (17). Treg-derived IL-35 induces the expression of PD-1 and other inhibitory molecules, and impairs T cell function in the tumor environment, thereby leading to tumor growth (35). Thus, blocking the PD-1/PD-L1 interaction can enhance the anti-tumor response by reducing the inhibitory activity of Tregs (109). Additionally, the IL-35 inhibitors can reduce the expression of PD-1 and other inhibitory cytokines and restore the anti-tumor immune activity of T cells. IL-35 participates in immunosuppression, and its expression is increased in PD-L1^+^ cells in NSCLC patients (17). Th1/Th2 cell and Th17/Treg cell ratios are unbalanced in NSCLC patients with CIP undergoing anti-PD-1/PD-L1 immunotherapy, leading to the increased secretion of IL-17A and IL-35 in BALF and serum (54). Thus, IL-35 inhibitors and PD-1/PD-L1 combination therapy may have a synergistic effect on NSCLC (17). Additionally, IL-35 can be used to assess the severity and improvement of CIP in NSCLC patients during immunotherapy (54). The roles of IL-35 in NSCLC are illustrated in **Figure 1**.

## IL-35, EGFR, and ALK

EMT is a complex process of phenotypic transition from epithelial cells to mesenchymal cells, while MET is the reverse transformation of the above phenotype (110). EMT plays a critical role in the occurrence, development, and treatment of resistant NSCLC (111, 112). Inflammatory cytokines promote the occurrence of EMT and advanced tumor progression. IL-35 has been shown to promote EMT and MET in different polarization states. In liver cancer, M1 macrophages secrete IL-35 to promote EMT through STAT3, and IL-35 leads to MET through M2 macrophage polarization, creating conditions for liver cancer progression (52). Interestingly, IL-35 secreted by tumor-associated macrophages can reverse EMT-promoted metastatic tumor colonization (113).

IL-35 inhibits PD-L1 expression in serum-starved ADC tumor cells (17). It has been shown to promote or reverse EMT in specific contexts, and EMT induces elevated PD-L1 expression in LC A549 cells (114). PD-1/PD-L1 blockade immunotherapy may be more effective in lung ADC patients with EMT phenotype (115). Interestingly, studies have shown that reversing EMT to a more epithelial phenotype contributes to increased responsiveness to immune checkpoint inhibitor therapy (116). Uncovering the complex mechanism of action of IL-35 on EMT and PD-L1 in NSCLC may help guide the treatment of LC.

Targeted drug therapy is a promising area for NSCLC, but drug resistance presents challenges for targeted therapy. EMT is associated with multiple targeted drug resistance mechanisms. Epidermal growth factor receptor (EGFR) tyrosine kinase inhibitor (TKI) is mainly aimed at the target of EGFR mutation gene in LC, but drug resistance is more common. EMT is a common mechanism of resistance to EGFR-TKI targeted therapy in LC (117). Reversal of EMT helps restore sensitivity to EGFR-TKI therapy in NSCLC patients (118). For anaplastic lymphoma kinase (ALK) rearranged NSCLC, ALK inhibitors are effective drugs. EMT is one of the mechanisms of drug resistance in ALK-TKI treatment of NSCLC patients (119). Additionally, hypoxia induced EMT resistance to ALK inhibitors with echinoderm microtubule-associated protein-like 4-ALK rearrangement (120). IL-35 can promote or reverse EMT in specific tumor microenvironments. Based on the complexity of the mechanism of NSCLC, IL-35 may regulate the molecular mechanism of NSCLC and the effect of targeted therapy drugs through various mechanisms, which is worth further exploration.

## Association of IL-35 With Baseline Inflammation During Immune Checkpoint Inhibition (ICI) Therapy

Immunotherapy including ICI against PD-1/PD-L1 is a promising treatment for LC. Available literature suggests that baseline systemic inflammatory markers and cytokines prior to treatment can predict ICI treatment effect and patient prognosis (121). Higher baseline inflammation is associated with poor prognosis (122, 123). Pre-treatment high inflammatory state and high levels of IL-6, and IL-8 cytokines are poor prognostic indicators of PD-1 inhibitor therapy, and high levels of IFN-γ are markers of good ICI treatment effect and prognosis (121). Additionally, high baseline levels of C-reactive protein, erythrocyte sedimentation rate, and procalcitonin during ICI treatment of NSCLC indicate poor prognosis (124). A series of adverse events, including CIP, can occur with ICI treatment. Pretreatment with COPD, high expression of PD-L1, and high baseline IL-8 levels are reported to be the risk factors for CIP (125). A study by Wang et al. demonstrated that serum IL-17A and IL-35 levels are significantly raised at the time of CIP diagnosis compared with those prior to treatment, and significantly decreased after clinical recovery. IL-17A and IL-35 were also increased during CIP in BALF. IL-35 was associated with changes in T lymphocyte subsets during the development of CIP. Thus, it is suggested that IL-35 may play a key role in the regulation of T-cell immune responses in CIP (54). However, at present, there are limited studies evaluating the effect of baseline systemic inflammatory markers combined with cytokine IL-35 on the treatment response and prognosis in NSCLC patients receiving ICI therapy. Further prospective studies are required to assess the molecular mechanism and clarify the role of IL-35 in ICI therapy. Relationship of IL-35 with baseline inflammation and immune modulation will help identify immunotherapy response effects and impact on patient outcomes.

The function of IL-35 is enigmatic, and its mechanistic studies in NSCLC are currently in the initial stage and have not yet reached the clinical trial stage. IL-35 has the potential to promote tumor development in NSCLC, and it has a central role in EMT, tumor resistance, and PD-1/PD-L1. At present, little is known about the kinetics of IL-35 during NSCLC chemotherapy or ICI treatment. Turnis et al. blocked anti-IL-35 and anti-PD1 in a tumor model, but showed no increase in tumor clearance, suggesting that they may in part be acting on the same pathway (35). Liao et al. established an extended model to explore the mode of action of anti-IL-35 therapy in a cancer model, and demonstrated that continuous injection was more effective than intermittent injection (10). Anti-IL-35 combined with ICI therapy provides a good prospect for the treatment of NSCLC.

## IL-35 Signaling in NSCLC

### IL-35 and Wnt/β-Catenin Signaling in NSCLC

The Wnt/β-catenin signaling pathway is conserved and closely related to embryonic development, homeostasis, and cancer (126), including NSCLC (127–129). The activity of this pathway depends on the cellular localization of β-catenin (127). Wnt-1 expression is positively correlated with the expression of c-Myc, cyclin D1, VEGF-A, MMP-7, and Ki-67 index, and with a poor prognosis of NSCLC (130, 131).

The expression of Foxp3 is upregulated in NSCLC and induces tumor growth and metastasis by stimulating the Wnt/β-catenin signaling pathway (132). Foxp3 induces EMT, tumor metastasis and growth, and reduces overall and recurrence-free survival, thereby worsening the prognosis (132). IL-35, produced by Foxp3-expressing Tregs, and the EBI3 subunit of IL-35, is a downstream target of Foxp3 (8). The serum levels of EBI3 are increased in LC patients, resulting in poor prognosis (20). Thus, IL-35 and Wnt/β-catenin may be useful diagnostic biomarkers for NSCLC. We hypothesize that IL-35 and the Wnt/β-catenin signaling pathway are closely related to the occurrence and development of NSCLC. However, additional studies are necessary to elucidate the underlying mechanisms.

### IL-35 and STAT Signaling in NSCLC

The IL-35 subunit EBI3 regulates the differentiation of T and B cells through the gp130-STAT3 pathway (133). STAT3 is activated in LC (134). It plays a dual role by inhibiting tumor cell growth and promoting metastasis in LC patients (135). Its increased expression promotes tumor progression, invasion, and metastasis (136), leading to poor prognosis (134, 137, 138). In coronary artery disease, IL-35 improves the function of B cells by suppressing the expression of IFN-γ and TNF by T cells, and the STAT3 signaling pathway may be involved in the suppression of T cell-mediated inflammation (139). IL-35 inhibits the differentiation and maturation of dendritic cells derived from monocytes *via* the STAT1/STAT3 and MAPK/NF-KB signaling pathways (140). In colorectal cancer, the expression of EBI3, gp130, and pSTAT3 is upregulated, and EBI3 promotes tumor growth through the gp130-STAT3 signaling pathway (141). In breast cancer, IL-35 induces the conversion of conventional T cells to iTr35 *via* the STAT1/STAT3 pathway (30). In rheumatoid arthritis, IL-35 inhibits angiogenesis through the Janus-related kinase (JAK)-STAT1 signaling pathway (142). However, whether IL-35 promotes angiogenesis in LC and other cancers is unknown. IL-35 is expressed in the trophoblasts of pregnant women and maintains maternal-fetal tolerance, probably *via* STAT1 and STAT3 (143). IL-35 protects against cardiac ischemia-reperfusion injury by reducing cardiomyocyte damage through the gp130-STAT3 signaling axis (144). These findings suggest that IL-35 and other factors in the STAT signaling pathway may serve as therapeutic targets for LC.

### Relationship Between IL-35 and JAK2-STAT6-GATA3 in NSCLC

EMT stimulates the metastasis of a variety of cancers (145, 146), such as colorectal cancer (147), bladder cancer (148), squamous cell carcinoma of the head and neck (149), and NSCLC (150, 151). Additionally, tumor-associated macrophages secrete IL-35 and promote metastatic colonization by regulating EMT through the activation of JAK2-STAT6-GATA3 signaling (113). However, the role of IL-35 and EMT in NSCLC is incompletely understood and warrants further research.

## Conclusion

IL-35 is an immunosuppressive factor strongly implicated in the development, treatment, and prognosis of cancers, including NSCLC. Based on the complexity of the mechanism of NSCLC, IL-35 may regulate the microenvironment and participate in immune escape and immunosuppression through various mechanisms, and more research is needed in the future. Thus, elucidating the role of IL-35 and its downstream signaling pathways in NSCLC may help guide individualized treatment and improve patient outcomes.

## Author Contributions

HD and YH drafted and revised the manuscript. WL, XL, and BS collected information and prepared the figures. PG conceived and designed the study. All authors contributed to the article and approved the submitted version.

## Conflict of Interest

The authors declare that the research was conducted in the absence of any commercial or financial relationships that could be construed as a potential conflict of interest.

## Publisher’s Note

All claims expressed in this article are solely those of the authors and do not necessarily represent those of their affiliated organizations, or those of the publisher, the editors and the reviewers. Any product that may be evaluated in this article, or claim that may be made by its manufacturer, is not guaranteed or endorsed by the publisher.

## Abbreviations

ADC, adenocarcinoma; AKI, acute kidney injury; ALK, anaplastic lymphoma kinase; ARDS, acute respiratory distress syndrome; CIA, collagen-induced arthritis; CIP, checkpoint inhibitor pneumonitis; COX-2, cyclooxygenase-2; DNP, diabetic neuropathic pain; EBI3, Epstein-Barr virus (EBV)-induced gene 3; EGFR, Epidermal growth factor receptor; EMT, epithelial-mesenchymal transition; FLS, fibroblast-like synoviocyte; Foxp3, forkhead box protein 3; gp130, glycoprotein 130; GATA 3, GATA binding protein 3; HNECs, human nasal epithelial cells; HUVECs, human umbilical vein endothelial cells; ICAM-1, intercellular adhesion molecule-1; ICI, immune checkpoint inhibition; IFN-γ, Interferon-gamma; ILC2, II innate lymphoid cells; IL-10, interleukin 10; IL-12, interleukin-12; IL-12Rβ2, interleukin 12 receptor β2 subunit; IL-35, interleukin-35; iNOS, Inducible nitric oxide synthase; JAK, Janus-related kinase; JNK, Jun N-terminal kinase; KDM3A, histone lysine demethylase 3A; LPS, Lipopolysaccharide; MAPK, mitogen-activated protein kinases; MDSCs, myeloid-derived inhibitory cells; MET, mesenchymal-epithelial transition factor; NK, natural killer T cells; NSCLC, non-small cell lung cancer; PBMCs, peripheral blood mononuclear cells; PD-1, programmed cell death protein; PD-L1, pIL-35, plasmid-IL-35; programmed cell death protein ligand 1; rIL-35, recombinant IL-35; rhIL-35, recombinant human IL-35; SCC, squamous cell carcinoma; SLE, systemic lupus erythematosus; STAT, signal transducer and activator of transcription; Tregs, regulatory T cells; TSLP, thymic stromal lymphopoietin; Th1, T helper type 1; Th2, T helper type 2; Th17, T helper type 17; TGF-β, tumor necrosis factor beta; TKI, tyrosine kinase inhibitor; TNF-α, tumor Necrosis Factor alpha; TTF, thyroid transcription factor; VCAM-1, vascular cellular adhesion molecule-1.
